# Improving Predictability and Effectiveness in Preventive Digital Health Interventions: Scoping Review

**DOI:** 10.2196/40205

**Published:** 2023-07-20

**Authors:** Keld Pedersen, Bjarne Rerup Schlichter

**Affiliations:** 1 Information Systems Department of Management Aarhus University Aarhus C Denmark

**Keywords:** mobile health, mHealth, digital interventions, adoption, implementation, prevention, physical activity, diet, mobile phone, scoping review, review

## Abstract

**Background:**

Lifestyle-related diseases caused by inadequate diet and physical activity cause premature death, loss of healthy life years, and increased health care costs. Randomized controlled trial (RCT) studies indicate that preventive digital health interventions (P-DHIs) can be effective in preventing these health problems, but the results of these studies are mixed. Adoption studies have identified multiple factors related to individuals and the context in which they live that complicate the transfer of positive results from RCT studies to practical use. Implementation studies have revealed barriers to the large-scale implementation of mobile health (mHealth) solutions in general. Consequently, there is no clear path to delivering predictable outcomes from P-DHIs and achieving effectiveness when scaling up interventions to reduce health problems in society.

**Objective:**

This research aimed to expand our understanding of how to increase the outcome predictability of P-DHIs by focusing on physical activity and diet behaviors and amplify our understanding of how to improve effectiveness in large-scale implementations.

**Methods:**

The research objective was pursued through a multidisciplinary scoping review. This scoping review used the PRISMA-ScR (Preferred Reporting Items for Systematic Reviews and Meta-Analyses extension for Scoping Reviews) as a guide. A comprehensive search of Web of Science and PubMed limited to English-language journal articles published before January 2022 was conducted. Google Scholar was used for hand searches. Information systems theory was used to identify key constructs influencing outcomes of IT in general. Public health and mHealth literature were used to identify factors influencing the adoption of, outcomes from, and implementation of P-DHIs. Finally, the P-DHI investment model was developed based on information systems constructs and factors from the public health and mHealth literature.

**Results:**

In total, 203 articles met the eligibility criteria. The included studies used a variety of methodologies, including literature reviews, interviews, surveys, and RCT studies. The P-DHI investment model suggests which constructs and related factors should be emphasized to increase the predictability of P-DHI outcomes and improve the effectiveness of large-scale implementations.

**Conclusions:**

The research suggests that outcome predictability could be improved by including descriptions of the constructs and factors in the P-DHI investment model when reporting from empirical studies. Doing so would increase our understanding of when and why P-DHIs succeed or fail. The effectiveness of large-scale implementations may be improved by using the P-DHI investment model to evaluate potential difficulties and possibilities in implementing P-DHIs to create better environments for their use before investing in them and when designing and implementing them. The cost-effectiveness of large-scale implementations is unknown; implementations are far more complicated than just downloading and using apps, and there is uncertainty accompanying implementations given the lack of coordinated control over the constructs and factors that influence the outcome.

## Introduction

Lifestyle-related diseases caused by inadequate diet and physical activity (PA) are a major problem in many societies, resulting in premature death, loss of healthy life years, and increased health care costs [1]. Facilitated by the widespread adoption of smartphones and wearables, preventive digital health interventions (P-DHIs) can present a more cost-effective approach to reach larger populations than traditional approaches [2,3]. However, randomized controlled trials (RCTs), adoption research, and implementation research indicate that predictable, positive outcomes from P-DHIs and the large-scale implementation of these solutions are difficult to achieve. Reviews of RCTs on P-DHIs that focus on PA and diet have revealed mixed results. Studies have reported no outcomes [4,5], small outcomes [6,7], outcomes that diminish over time [8], limited evidence for positive outcomes [9-11], positive outcomes for some individuals in some settings [12], mixed outcomes [13-15], promising results [16,17], and effectiveness of P-DHIs [18-22]. Beyond RCT studies, adoption research [23-26] indicates a range of factors related to the technology, individuals, and the context in which they live that complicates the transfer of results achieved in RCT studies to other persons in general. Furthermore, implementation research [27,28] identifies implementation barriers in health care organizations and society that hinder large-scale implementation of mobile health (mHealth) solutions, including the P-DHIs studied in this review.

Although low methodological quality may account for some of the uncertainty regarding the outcomes reported by RCT studies [14], the mixed results and adoption and implementation difficulties are unsurprising from an information systems perspective. We know that organizations experience quite different outcomes when investing in similar IT as the outcomes depend on many factors other than the IT [29]. Richardson and Zmud [30] emphasize that “The salient question, then, is not ‘Does IT pay off?’ but rather ‘Under what conditions does IT pay off?’” This is the core question behind this research as well. Specifically, this study investigated how to increase the predictability of outcomes of P-DHIs focusing on PA and diet behaviors and how to improve the effectiveness of large-scale implementations of P-DHIs.

This research was conducted as a scoping review as evidence regarding how to improve predictability and effectiveness from large-scale implementations of P-DHIs is unclear and a broad multidisciplinary understanding of this issue is needed. Investigating these issues is crucial for successfully exploiting P-DHIs as part of large-scale public health initiatives that demand both predictability and effectiveness. The development of the P-DHI investment model addresses these issues. The model is based on general constructs from information systems theory known to strongly influence outcomes from IT investments and factors closely linked to the specific use of IT in P-DHIs. The factors studied in this review relate to the adoption of P-DHIs, behavior change supported by P-DHIs, and the implementation of P-DHIs in society, thereby influencing P-DHI outcomes.

The concept of *health care organization* in this study references a wide variety of organizations, including public and private sector organizations at national, regional, and community levels that are engaged in public health and provide preventive initiatives.

The concept of *community* refers to both the physical community in which a person lives and their social community, which may include web-based social networks established through social media applications.

*Tailoring* refers to the process of adapting a P-DHI to the specific context (including specific persons) for which its use is intended. Similar research also uses *customization*, *individualization*, and *personalization* to name this process. *Tailoring* is used in this paper as it is a broader concept.

The study of P-DHIs in this review includes the use of smartphone apps and wearables as a key component, as well as additional resources accessed through the apps (eg, web-based social networks with other persons facing similar health-related concerns or knowledge provided by health care experts). A P-DHI is perceived as consisting of both IT (the IT investment) and additional investments (the non-IT investments) made to implement and benefit from the IT.

## Methods

### Overview

This research is based on a systematic multidisciplinary scoping literature review [31,32] using the PRISMA-ScR (Preferred Reporting Items for Systematic Reviews and Meta-Analyses extension for Scoping Reviews) as a guide (Multimedia Appendix 1). The method used in this review included the steps outlined in Textbox 1.

**Method used in this review.**

**Identifying relevant literature streams**

- Given the multidisciplinary nature of the factors influencing the outcomes of preventive digital health interventions (P-DHIs; eg, self-efficacy [33], software quality [34], and factors in the context [35]), it was appropriate to explore a broad range of literature streams.

**Identifying articles within the literature streams**

- This was done primarily by identifying high-impact theoretical models and literature reviews and secondarily by identifying individual empirical studies.
- Analyzing articles, coding, and categorizing the constructs and factors from the articles and developing the P-DHI investment model

### Identifying Relevant Literature Streams

The choice of literature streams and articles was guided by the concerns outlined in Textbox 2.

**Concerns that guided the choice of literature streams and articles.**

**Which overall constructs influence the outcomes of using IT in general?**

- The information systems literature focusing on value creation from IT was reviewed to identify these constructs.

**Which factors influence the outcomes of prevention initiatives in general?**

- To address this question, articles describing the most frequently used theoretical models in the public health intervention literature were reviewed.

**Which specific factors influence preventive digital health intervention (P-DHI) outcomes?**

- The articles included to address this question related to (1) adoption (factors influencing the degree to which persons adopt P-DHIs), (2) health outcome (factors related to P-DHI effectiveness in terms of influencing behavior and improving health), and (3) implementation (factors influencing the degree to which it is possible to implement P-DHIs in health care organizations and society).

### Identifying Articles: Eligibility Criteria

Textbox 3 presents the eligibility criteria for articles in the literature streams.

**Eligibility criteria.**

**General eligibility criteria for all literature streams**

- Peer-reviewed journal articles
- Articles written in English
- Articles published before January 2022

**Eligibility criteria for information systems articles**

- Review articles (including models based on reviews) focusing on IT business value creation
- Articles that identify constructs that influence outcomes of IT in general
- Articles focusing specifically on technologies or specific industries not related to the research question were excluded.

**Eligibility criteria for theoretical models used in public health**

- Articles describing the most frequently used theories to research health-related behaviors within public health
- Articles that identify factors that influence health-related behaviors and behavior change
- In 2015, the following were identified as the most frequently used theories [36]: the transtheoretical model of change (used in 91/276, 33% of the identified articles), the theory of planned behavior (36/276, 13%), social cognitive theory (29/276, 10.5%), the Information–Motivation–Behavioral Skills Model (18/276, 6.5%), the Health Belief Model (9/276, 3.3%), self-determination theory (9/276, 3.3%), the Health Action Process Approach (8/276, 2.9%), and social learning theory (6/276, 2.2%). Even though the socioecological model is not among the most frequently used models, it was included as it offers insight into the relationship between the context, behaviors, and health not provided by the other theories.

**Eligibility criteria for mobile health (mHealth) articles**

- Articles identifying factors influencing the adoption of preventive digital health interventions (P-DHIs)
  Reviews and empirical articles focusing on the adoption and use of P-DHIs, including interventions focusing on physical activity (PA) and diet
- Articles identifying factors that influence P-DHI health outcomes in terms of influencing behavior and health
  Reviews of mHealth articles focusing on outcomes reported in PA or diet randomized controlled trial studies
  Articles that identified factors influencing the outcome in terms of behavior change and health (such as the inclusion of behavior change techniques in the design)
  Articles suggesting standards and taxonomies describing factors that influence the outcome in terms of behavior change and health
- Articles identifying factors influencing the large-scale implementation of P-DHIs
  Only review articles focusing on the implementation of mHealth were included. Given that many of the barriers are assumed to be independent of the specific purpose of the mHealth solution, this literature review included articles that addressed mHealth implementation in general as well as articles specifically focusing on prevention related to diet and PA.
- Articles focusing on very specific issues, such as specific diseases (eg, sexual health), a very specific geographical area, or a very specific technology (eg, blockchain), were excluded.

### Identifying Articles: Search Strategy

Search strategies for each of the literature streams were developed using search strings based on keywords, as described in Textbox 4.

Web of Science was used because of the multidisciplinary nature of the literature review. PubMed was used to specifically search for mHealth articles, thereby removing the risk of relevant mHealth articles not being found through Web of Science. Google Scholar was used to identify highly cited theoretical models used in public health (Textbox 4) based on the study by Davis et al [36]. The search strings used in Web of Science and PubMed were tested in pilot searches. Keywords were added and removed to determine whether a broader search would include relevant articles that were not identified using narrower search strings. For example, “fitness app” was added to include a higher number of relevant articles. In the mHealth adoption search string, the term “review” was removed as including this keyword excluded significant insights in this literature stream.

**Search strings.**

**Information systems articles**

- Search string: TS=((“it” OR “information technology” OR “is” OR “information system”) AND “business value” AND review)
- Both “IT” and “IS” (information system) were included as especially older articles use the concept of “information system,” not “information technology.”
- Database: Web of Science

**Theoretical models used in public health**

- Keywords: “Transtheoretical Model of Change,” “Theory of Planned Behavior,” “Social Cognitive Theory,” “Information-Motivation-Behavioral-Skills Model,” “Health Believe Model,” “Self-determination Theory,” “Health Action Process Approach,” “Social Learning Theory,” “Socio-Ecological Model”
- Database: the keywords were used in individual searches in Google Scholar to identify highly cited articles describing the models.

**Mobile health articles**

- Articles identifying factors that influence adoption and use of preventive digital health interventions (P-DHIs)
  Search string: TS=((mHealth OR m-health OR “mobile health” OR smartphone OR “mobile app*” OR “mobile application*” OR “fitness app” or “diet app”) AND (“physical inactivity” OR overweight OR obesity OR nutrition OR diet OR “physical activity” OR fitness OR prevent* OR “chronic disease”) AND (adoption OR “technology acceptance model” OR TAM* OR “unified theory of acceptance” OR UTAUT* OR “use of technology” OR “IS success model”))
  This search string did not include the concept “review” as initial searches using this concept returned too few articles and left out significant contributions.
- Articles identifying factors that influence P-DHI outcomes in terms of influencing behavior and health
  Search string: TS=((mHealth OR m-health OR “mobile health” OR smartphone OR “mobile apps” OR “mobile applications” OR “fitness app” or “diet app”) AND (prevent* OR “behavioral change” OR “behavior change”) AND (“physical inactivity” OR overweight OR obesity OR nutrition OR diet OR “physical activity” OR fitness) AND (review))
- Articles identifying factors influencing the large-scale implementation of P-DHIs
  Search string: TS=((mHealth OR m-health OR “mobile health” OR “fitness app” or “diet app”) AND implement* AND review)
  The search criteria were broader than in previous searches as it was assumed that many implementation issues are general and not specific to apps focusing on diet and physical activity.
  Database: Web of Science and PubMed. Searches in Web of Science used TS (topic). Searches in PubMed used “Title/Abstract”. The same keywords were used in both databases.

### Identifying Articles: Screening and Eligibility

**Figure 1:**
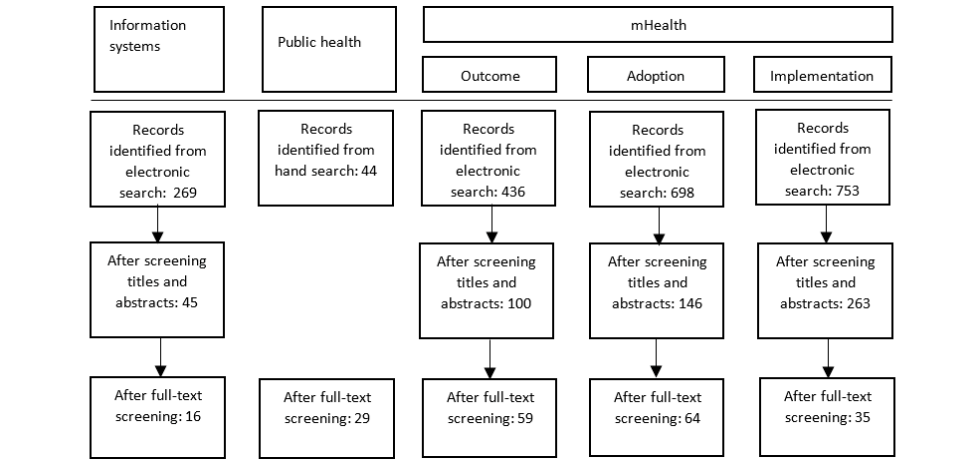
The screening process. The mobile health (mHealth) articles identified from the electronic search contained some duplicates across the 3 searches. The numbers in the after screening titles and abstracts section in the diagram are the numbers of unique articles.

The search results from Web of Science and PubMed were downloaded to Microsoft Excel (Microsoft Corp). There was a substantial overlap between the mHealth search results from Web of Science and PubMed. The number of records identified from the electronic search reported in Figure 1 was after the removal of duplicates. Both authors screened the articles independently of each other, and subsequently, the results were compared and discussed.

Titles and abstracts were screened against the eligibility criteria (Textbox 3). The excluded articles were labeled with reasons for exclusion. Only articles clearly outside the scope of interest were excluded in this step. The inclusion and exclusion criteria were updated during the screening process. A total of 554 articles were extracted for full-text screening. The same inclusion and exclusion criteria were used for full-text screening. During full-text screening, additional papers were included based on a backward search using Google Scholar. A total of 203 articles met the inclusion criteria. Several articles identified and described the same factors. In particular, adoption research reported many identical factors as the studies were based on the same information system adoption models. For example, social influence (subjective norms) was emphasized in many adoption research papers. To reduce the number of references, not all articles that emphasized, for example, social influence were included in the references. The same inclusion and exclusion criteria were used for title and abstract and full-text screening. Owing to the nature of the review, bias concerns were not used to exclude articles. Figure 1 illustrates the screening process.

### Data Charting Process: Analyzing Articles, Extracting, and Structuring Constructs and Factors

The identified articles were imported into NVivo (QSR International) and analyzed, and the constructs and factors identified in the articles were coded in an iterative process.

The first step was to use information systems theory to identify constructs that influence outcomes from investments in IT in general. For example, “context” was identified as a key construct.

The second step was to identify and categorize factors from the public health and mHealth literature influencing (1) adoption, (2) outcomes (eg, behavior change and health) specifically from P-DHIs, and (3) the possibilities for large-scale implementation. For example, multiple sources within the public health and mHealth literature emphasized the importance of “social influence” for both adoption and behavior change.

In the third step, the factors from the public health and mHealth literature were categorized using the key construct identified in information systems theory—IT and non-IT investments establishing P-DHIs, the context in which P-DHIs are implemented and used, and the lag effects that influence when outcomes from P-DHIs are realized. For example, “social influence” was categorized as a part of the “context.” Additional lower-level constructs were included as well (eg, specific parts of the context)—information systems theory is concerned with organizational processes, not processes in a person’s life, and the context for health-related behavior change includes a person’s changing behavior and the community in which they live.

Finally, the P-DHI investment model was developed based on the general information systems constructs and related factors from the public health and mHealth literature.

One coder (the first author) coded all the articles, making it easier to ensure consistency but introducing validity and reliability concerns. Another coder (the second author) independently coded approximately 10% of the articles (22/203, 10.8% of the articles), and the coding was subsequently compared to reduce validity and reliability concerns. Only minor discrepancies in coding were identified, discussed, and resolved. The level of uncertainty and subjective interpretation when coding the text from these articles was low. Regarding the information systems articles, constructs influencing the outcome were coded using the concepts (eg, lag effects) in the articles. Regarding the public health and mHealth articles, the factors in these models (eg, perceived self-efficacy) influencing behavior change and health were coded using the concepts from the articles. During the coding process, fewer and fewer new concepts were added because of the conceptual consensus across the articles. Subsequently, additional categories were included to group the factors and establish a more general understanding (eg, some factors related to the capabilities of individuals).

## Results

### Overview

**Figure 2:**
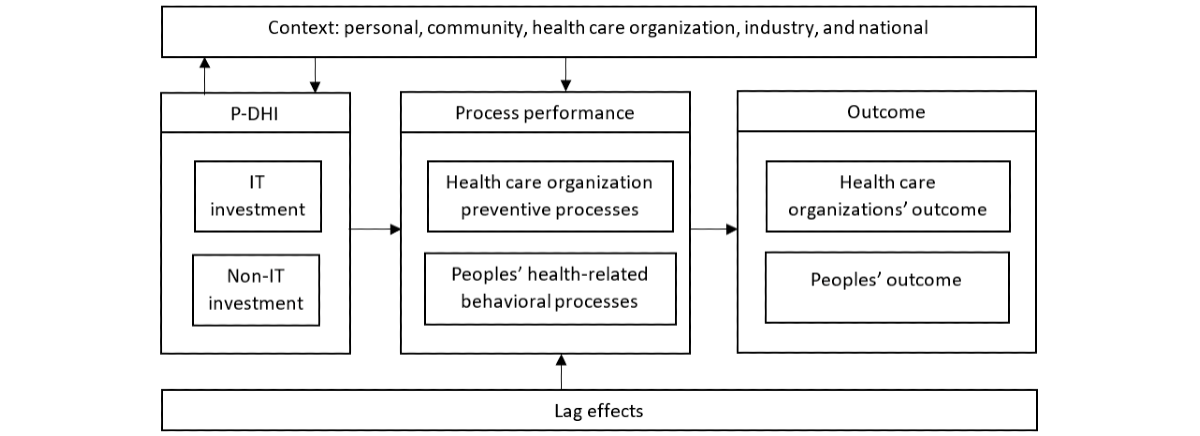
The preventive digital health intervention (P-DHI) investment model.

The results in terms of the P-DHI investment model are illustrated in Figure 2. The model illustrates how outcomes from using P-DHIs are created and, consequently, how predictability and large-scale effectiveness might be improved. First, the P-DHI investment model is explained. Second, the constructs and factors in the model are presented.

### The Model

The P-DHI investment model is based on the constructs used in information systems theory to explain outcomes from the use of IT [29,37,38], specifically based on the model by Schryen [39]. The basic logic is that an outcome is created, in this case, for example, weight loss, by changing the performance of the processes involved. In the case of weight loss, it is processes in health care organizations delivering preventive health care services and processes in terms of individual health-related behaviors associated with weight loss. Changes are achieved using a combination of IT and non-IT investments. IT investments are investments in apps and wearables integrated with health care systems such as a database collecting data from individual P-DHI users. Non-IT investments are additional investments in changes in health care organizations, the services they provide, and society in general necessary for delivering prevention using the P-DHIs, as well as additional investments made by P-DHI users to change behavior, such as investments in fitness equipment, time, and energy needed for behavior change.

The P-DHI should be tailored to match the characteristics of the context, and the context may support or complicate process changes within health care organizations and in P-DHI users’ lives. For example, health care professionals might resist using this kind of technology, and P-DHI users might experience a lack of social support or find it difficult to change their behaviors for other reasons. Consequently, the P-DHI might provide support for overcoming barriers and exploiting resources in the context. Lag effects, for example, learning how to use P-DHIs, can delay how long it takes to create positive outcomes. The following sections explain the factors in various parts of the model that influence the outcome.

### P-DHIs: IT Investment

The 5 factors in Textbox 5 are important as they are closely related to the improvement of people’s health-related behaviors and, thereby, the outcome.

**IT investment factors that influence the outcome of preventive digital health interventions (P-DHIs).**

1. Complying with software quality requirements: the degree to which the P-DHI app complies with basic software quality requirements and is integrated with other relevant IT systems
2. Tailored to the context: the degree to which the P-DHI app is tailored to an individual’s context and personal needs
3. Developing personal capabilities: the degree to which the P-DHI app supports the development of the personal capabilities needed for behavior change using a P-DHI app
4. Behavior change support: the degree to which the P-DHI app provides theory-based behavior change support
5. Provides additional personal help: the degree to which the P-DHI app provides additional help through access to web-based social networks and health care professionals during the behavior change process

The logic behind the first 5 factors is that, if a P-DHI app lacks basic software quality characteristics, such as being easy to use, it will reduce adoption and use, and to provide relevant support for the individual users, it needs to be tailored to the context in which they live and their individual needs. Using P-DHIs to change health-related behaviors requires behavior change capabilities in general as well as capabilities related to specific behaviors (eg, exercise-related capabilities) and capabilities in the use of this kind of technology. If a person lacks these capabilities, they need support from the P-DHI to develop them. The actual behavior change process is best supported using behavior change theory, such as the transtheoretical model of health behavior that supports the entire behavior change process and behavior change techniques (BCTs) that support individual steps in this process. During the behavior change process, there might be a need for web-based personal help from health care professionals and peers taking part in similar change processes. The remainder of this section describes the theoretical foundation behind these 5 factors.

The software quality requirements can be summarized as being easy to use [21,26,34,40-46], engaging and interesting [34,47-49], trustworthy (eg, in terms of being credible [34,50] and secure [26,27,46,51]), technically robust [26,52], and affordable [27,47,53-57]. The strong focus on usability is not surprising given that perceived ease of use [58] is influential on a person’s intention to adopt P-DHI apps [59]. However, the review by Milne-Ives et al [11] found no relationship between usability and app effectiveness, that is, although usability ratings were high in the reviewed RCT studies, app effectiveness remained low. Being easy to use is important for adoption and use but not enough to create positive outcomes.

P-DHI apps should be integrated with dependable and locally adapted food databases [56] and other relevant apps [26]. In particular, mHealth implementation research emphasizes the need for integrating apps with health care information systems used by health care professionals [28,35,60-66].

Similar to any other software, P-DHI apps should be user-centered and tailored to the context [22,26,40,42,46,67-77]. For example, we know that P-DHI app use of BCTs should be tailored as their efficacy depends on users’ sociodemographic [10,78] and psychosocial factors [10]. However, tailoring includes adapting the P-DHI app to the entire context. Overcoming barriers [79-84] and exploiting specific resources in the context [80,85-88] are vital parts of behavior change. The following section about the context describes contextual factors that should be considered for tailoring. Being user-focused and developing tailored P-DHI apps seems quite complicated compared with the development of IT systems to be used within organizations because of the number of users, variations across users, and variations in users’ contexts.

Behavior change requires the capabilities to change and perform new behaviors and belief in these capabilities (perceived self-efficacy) [33,64,80,89,90]. This review identified 3 different types of capabilities: capabilities related to behavior change in general, such as capabilities to self-regulate [91]; capabilities specifically related to the execution of new behaviors (eg, to exercise or prepare healthy food) [90]; and capabilities to use technology as a part of health-related behavior change, for example, in terms of eHealth literacy [4]. Consequently, P-DHI apps should support the improvement of people’s capabilities for health-related behavior change as well as their level of perceived self-efficacy if needed. This can be accomplished in many different ways, such as providing guidance [44,83,84], opportunities for practice [83,84], experience-based learning [33,75,81,92], social models [88,91,92], and mastery experiences (graded tasks) [83,84,91]. Wester et al [4] suggested identifying and improving low eHealth literacy before the actual behavior change intervention to make interventions more successful for people with low socioeconomic status.

The core of a P-DHI app is, of course, the features that support the behavior change process. Research indicates that theory-based P-DHIs are more effective [26,42,46,81]. This review identified 4 types of behavioral theories that seem especially relevant. The first type of theory is the models (transtheoretical model of change [89] and Health Action Process Approach [79]) that cover the entire behavior change process and divide the process into stages; the second type is the taxonomies that describe the specific BCTs (eg, goal setting) to be used during the stages [84]; and the third type of theory focuses on specific factors, such as perceived self-efficacy, that have a large impact on the possibilities for successful behavior change [33]. The last kind of theory, the socioecological models, focuses on how the context influences health-related behaviors, behavior change, and health. For example, how the physical environment influences PA behaviors [85,93-95] and how the food environment influences diet behaviors [96,97]. The implication of being “theory-based” is that a P-DHI app should provide different kinds of support depending on the person’s current stage in the behavior change process and that the functionality should support the use of BCTs, attempt to influence the key factors such as perceived self-efficacy positively, and be context aware and help users exploit resources and overcome barriers in the context. There is some uncertainty about which BCTs to use [8,10,20], how best to combine them [8,10], and how best to implement them within apps [11,98]. Some RCT reviews [11,21] reported no association between the number of BCTs applied in apps and app effectiveness, whereas another review of P-DHIs focusing on PA reported that initiatives failed for people with low socioeconomic status irrespective of the BCTs used [4]. The inclusion of BCTs in apps is important but not sufficient to achieve effectiveness [11].

During behavior change, 2 different sources might provide additional support that complements the support provided by the P-DHI app functionality—support from social networks and health care professionals. Providing access to social networks through P-DHIs is generally perceived as beneficial [15,99]. Social networks potentially provide access to many resources that we know influence the adoption of P-DHIs, continued use of P-DHIs, and behavior change—social models [84,88,92], social influence [26,53,59,84,100-102], social support [15,80,85,88,89,103], social comparisons [12,83,84], and social connectedness and a feeling of relatedness to a community [45,104,105]. Although social networks can provide access to many resources and reportedly increase engagement with PA interventions [106-108], research findings [16,26,44,109-111] regarding the benefit of social networks and features are mixed. Some discrepancies may be attributed to differences in social comparison orientation [109]. Koönig et al [26] reported that social features serve to motivate some, whereas participation in competitions or observing others’ success is demotivating to others. Excessive competition in social networks [15] and lack of alignment of behavioral goals between the person attempting to change their behavior and other participants in the network [16] can negate the positive effect of social networks.

Combining P-DHI app use with support from health care professionals seems to offer greater effectiveness than stand-alone interventions [10,51,64,112], and therefore, it may be beneficial to provide some degree of access to health care professionals through the P-DHI. Some uncertainty remains regarding the relative contribution of coaching delivered by health care professionals and the app itself [15,18]. Using apps as stand-alone solutions increased PA in some studies but failed to do so in others [15].

### P-DHIs: Non-IT Investment

Generally, non-IT investments are investments in organizational changes that are required to benefit from IT investments [39,113]. This study used a broader understanding of non-IT investments as the purpose of P-DHIs is to change individuals’ behaviors. The first 3 factors in Textbox 6 influence the performance of health care organizations’ preventive processes, whereas factor 4 influences the improvement of people’s health-related behaviors.

**Non-IT investment factors that influence the outcome of preventive digital health interventions (P-DHIs).**

1. Integrating the use of P-DHIs into health care organizations: the degree to which the use of P-DHIs is properly integrated into health care organizations’ preventive processes
2. Recruiting and engaging P-DHI users: the degree to which health care organizations succeed in recruiting and engaging P-DHI users
3. Providing additional services for P-DHI users: the degree to which health care organizations provide additional services, such as workshops that support behavior change
4. P-DHI users’ investments: the degree to which P-DHI users invest the required resources in terms of engagement and motivation, time, and money, among others, for changing their behavior

The logic behind the factors in Textbox 6 is that, even if a highly sophisticated P-DHI app is bought or developed, it does not produce positive outcomes for society before it is properly integrated into health care organizations’ processes focusing on preventing chronic diseases in the population. Furthermore, it requires investment in targeted campaigns that recruit and engage people in society who are at risk of developing chronic diseases caused by lifestyle-related problems. Finally, positive outcomes require more from P-DHI users than downloading an app; they require considerable engagement, time, and money from the user. The remainder of this section describes the theoretical foundation behind the aforementioned 4 factors.

Similar to any other technology, the integration of P-DHIs in health care organizations requires organizational changes to succeed. The literature specifically on the implementation of P-DHIs is very sparse, but the literature on mHealth implementation in general emphasizes the same kind of changes as the implementation of IT in general (eg, new incentives [43,62], policies [43,67], ways of working [28,60-63,114] and collaborating [40,54], and new capabilities [27,63]). Integration also includes establishing the facilities needed for providing training and support for using P-DHI apps to change behavior [49,66,102,115,116]. The suggested ways to implement these organizational changes are also similar to what we know from the implementation of other types of IT systems—formulating implementation strategies [62,67], managing organizational resistance [67], and training internal users [40,61,62,67,117].

In addition to these internal changes, implementation involves choosing and using engagement and recruitment strategies, such as promotion and marketing campaigns and clinical endorsement [41]. Both the transtheoretical model of change [89] and the Health Action Process Approach [79] distinguish between initial stages, in which potential users of a P-DHI do not even acknowledge that they need to change their behavior or are uncertain about engaging in behavior change even though they acknowledge the need, and later stages, in which actual behavior change actions are executed and potential users attempt to maintain behaviors. Therefore, different campaigns are needed for potential users depending on their stage in the process.

Research indicates that multicomponent interventions involving additional services are generally more effective than stand-alone app interventions [10,18]. The RCT reviews studied in this paper described additional services in the included RCT studies, such as workshops and group sessions [5,8,18], individual education and coaching sessions [4,8,15,18,19], mindfulness sessions [8], personalized feedback from health care professionals [19,21], and motivational interviewing [19]. Kozik et al [118] suggested that training of individual persons should be tailored to the context in terms of addressing inequity issues through the provision of special onboarding sessions for advanced-age and low-education populations. This kind of personal support from health care professionals creates scalability problems and may make P-DHIs less attractive from an economic perspective.

We know little about the non-IT investments for P-DHI users and how they affect the outcome. Collecting data on this type of investment is not addressed in the P-DHI research reviewed in this study. From the behavior change theory and models [79,89] and the BCT taxonomy [84] that describes the behavior change process and specific activities (eg, self-monitoring and regulation), we can deduce that P-DHI users must invest considerable time, energy, mental and physical resources, and money to motivate themselves, plan and execute behavior change activities, bounce back from setbacks, and rearrange their life and context so that they support the new behaviors.

### The Context

Generally, the same kind of IT system can lead to different results in different contexts [29,37-39], and this also applies to P-DHIs. The socioecological theory emphasizes how the context influences behaviors and behavior change [88,119], the behavior change wheel [120] emphasizes the importance of persons’ opportunities for successful behavior change, and Han and Lee [22] report that the use of P-DHIs in different situations for different persons may lead to varied outcomes.

A wide range of contextual factors influences how P-DHIs should be designed and tailored, and the factors in Textbox 7 influence the outcome of P-DHIs.

**Contextual factors that influence the outcome of preventive digital health interventions (P-DHIs).**

1. Individual users’ characteristics: the degree to which the P-DHI matches individuals’ current stage in the behavior change process and their characteristics and influences these characteristics positively
2. Community-level characteristics: the degree to which the P-DHI supports behavior change in the P-DHI user’s specific community
3. Health care organization readiness: the degree to which the necessary resources are in place to support the implementation of P-DHIs in health care organizations
4. Health care sector requirements: the degree to which P-DHIs comply with core health care sector requirements
5. National-level support for the use of P-DHIs: the degree to which national-level funding, policies and regulations, and technological infrastructures support the use of P-DHIs

The logic behind these 5 factors is that P-DHI users who are different and live in different communities that provide different barriers and possibilities for behavior change need diverse kinds of support. Even if the P-DHI matches these characteristics and provides the right support, success still depends on health care organizations being ready to implement P-DHIs. Furthermore, to become a part of health care services, P-DHIs need to comply with the formal requirements that we expect from health care services, such as being evidence-based, and large-scale implementations require national-level support and sufficient technological infrastructures in society. The remainder of this section describes the theoretical foundation behind these 5 factors.

P-DHI users at dissimilar stages in the behavior change process need diverse kinds of support. The main difference is between the initial stages, in which potential users are developing intentions to change, and later stages, in which they attempt to change or maintain the behavior [79,89]. Some of the personal characteristics that the P-DHI needs to be designed for and tailored toward are unmodifiable in the sense that they cannot be changed as a part of the behavior change process. Demographics and socioeconomic status influence individuals’ acceptance of and use of P-DHIs [17,26,42,55,57,118,121-124] and the outcome of interventions [4,12,125,126]. The degree to which a P-DHI app is consistent with personal values [116] and culture [42,121,127] also influences individuals’ acceptance.

Other characteristics are modifiable, and the P-DHI should attempt to influence them to improve the possibilities of successful behavior change. These characteristics are related to the users’ intentions for behavior change, their capabilities for behavior change, and their situation in life. Potential P-DHI users’ level of health consciousness [128-130], perception of their own health and health risks [80,82,124], expectations regarding the outcome of changing behaviors [80], attitudes toward new behaviors [131], and self-efficacy beliefs have a strong influence on intentions for behavior change. The required capabilities were described in the previous section. Current life situations include the degree to which P-DHI users face issues such as stress [91], feeling tired [91], being depressed [91], temptations to deviate from new behaviors [89], lack of time [26,44,85], or competing priorities that make behavior change difficult [41].

The resources available in a specific context have a large impact on health-related behavior, behavior change, and health [80,85-88]. For example, we know that access to community-level health care resources is important [86].

The social context in terms of social influence is important for the general acceptance of P-DHI apps [23], the intention to adopt these apps [26,132,133], the intention to use these apps [25,49,53,100,134], the actual use of these apps [103], and the continued use of these apps [24,44,50,111,135]. The physical context influences both food intake [96,97] and PA [85,93-95,136]. As previously described, P-DHIs should help P-DHI users overcome barriers and exploit community-level resources. Tonkin et al [76] suggested that P-DHI apps could provide information about local stores offering healthy food options and assist in creating a healthier food environment, might help find appropriate fitness partners [77], and generally help rearrange the context to support new healthy behaviors [81].

As previously described, under non-IT investments, the widespread use of P-DHIs requires changes to preventive processes within health care organizations. We know little specifically about the implementation of P-DHIs, but we do know that the successful implementation of mHealth in general requires adequate management resources [62], financial resources [60-63,67,117], and IT resources [28,35,43,62,137]. Furthermore, different types of organizational capabilities (eg, project management capabilities) are needed when implementing these solutions [27,28,35,51,60,63,67,138]. One of the most important issues is health care professionals’ attitudes toward these solutions [14,28,63], their outcome expectancies [14,40,43,62,66,139], their resistance to change [28,63,67], and their perception of the organization’s readiness to use mHealth [62,138]. Some sources mention that difficulties in implementation can be attributed to health care organizations’ relatively slow adoption of new technologies such as mHealth [67,118], their organizational culture [35,67] and norms [140], and frequent budget deficits [62].

P-DHIs should comply with the requirements posed by the health care sector in general. However, there is generally a lack of regulation (eg, Food and Drug Administration approval or other kinds of certification) of mHealth apps [63,65,118,141], there are differences in medical and clinical practices across state or country lines that can complicate the use of mHealth apps [60], and there is a lack of evidence regarding the effectiveness of mHealth in practical use that one would normally expect from elements in health care services [63].

Government support for the use of mHealth [35,40]; government involvement [27,57]; funding [27,40,57]; and mHealth policies, strategies, and guidance [27,40,57,61,63] are important for P-DHIs to be used as a central part of national public health initiatives. Furthermore, P-DHIs and other mHealth solutions require widespread access to mobile technology [27,35,40,41,43,74,118,122,138,142] and reliable technological infrastructures [14,27,43,54,63,137,138,142,143].

### Outcomes, Process Changes, and Lag Effects

The outcomes for health care organizations and individuals are divided into 2 categories: tangible and intangible. The outcomes listed in this section are the possible outcomes mentioned in the reviewed literature. We know that P-DHIs in some cases change PA and diet behaviors [7,8,10,13,16-18,21,22], and we know that the behavior changes from using P-DHIs positively influence health [7,12,13,18,19]. On the basis of this review, little is known about the impact on intangible outcomes such as P-DHI users’ capabilities for long-term health management. Similarly, we know little about the impact on health care organization outcomes (eg, in terms of reduced costs).

Tangible outcomes include improved health-related behaviors and health, other impacts on individuals (eg, improved convenience), changes to health care professionals’ work (eg, workload), and impact on health care organizations (eg, improved cost-effectiveness). Improved health-related behaviors and health are, quite naturally, emphasized in the reviewed literature [6,11,14,21,22,117]. Health-related outcomes can also involve improved appearance, regaining past fitness, or complying with job requirements [111]. There are also more practical outcomes for individuals, such as easier access to health care [14,16,27,62,117], improved convenience [14,51] and communication [14,27,117], and lower costs for individuals using mHealth [27,117]. Although these outcomes are positive, P-DHI apps pose a risk of discriminating against people with low socioeconomic status [4,125,126] on the wrong side of the “digital divide” [42,126,144,145]. There are also concerns about P-DHI apps leading to unhealthy behaviors such as food choices based on ease of registration within the app, extreme calorie restriction, and eating disorders [26].

Health care organizations might experience improved cost-effectiveness [14,16] by reaching more persons at a lower cost [59], using more scalable health care services [16], and improving patient care [62]. P-DHIs may influence several aspects of the health care professional experience both positively and negatively. The reviewed literature mentioned aspects such as workload [7,14,43,62,66,139], record maintenance [14], job security [40,62], efficiency, job autonomy, and effectiveness [62].

Intangible outcomes include increased awareness about health, increased motivation for changing health-related behaviors, external acknowledgment, psychological development and well-being in general, and improved health and behavior change capabilities. There are several positive intangible outcomes that may increase individuals’ possibilities for long-term health outcomes: increased awareness of health-related issues [26,74,82,89,91,139], increased motivation for changing health-related behaviors [12,26], acknowledgment from social networks [91], psychological development and well-being [12], increased capabilities (eg, more knowledge about health [26,80,146] and behavior change–related skills [26]), and improved self-efficacy [7,12]. Furthermore, users may experience greater empowerment and improved daily life autonomy [7]. On the negative side, attempts to change behavior can also lead to negative feelings such as guilt, disappointment, anxiety when failing, or feeling neurotic about one’s own body image [26]. mHealth might increase social isolation among older adults, who might prefer direct in-person contact with health care professionals [17].

Owing to lag effects, outcomes from IT investments are generally not realized immediately [39]. IT investments can even lead to worse performance in the interim because of learning-by-doing effects [147]. Stephenson et al [8] found a decrease in behavior changes from P-DHIs, specifically reduced sedentary behavior (in RCT studies), over time, whereas Emberson et al [12] reported that, with regard to PA (in RCT studies), interventions of longer durations led to better results than those of shorter durations. The meta-analysis by Moönninghoff et al [148] found that, although the effects of PA interventions were maintained in the long term, the size of the effect diminished over time. Generally, we lack knowledge about the long-term effectiveness [6,13,148] and cost-effectiveness of P-DHIs that promote PA and sedentary behavior changes [6]. There is no research among the studies in this review specifically exploring lag effects in the context of time elapsed between the implementation of a large-scale P-DHI and changes to process performance in health care organization prevention processes and people’s health-related behaviors being realized. In addition, there is no research explicitly addressing the factors that contribute to lag effects.

### Using the Model in the Prevention of Lifestyle-Related Health Problems

The P-DHI investment model and its 14 factors can be used by health care organizations when considering, designing, and implementing P-DHIs.

When considering using a P-DHI, the P-DHI investment model can be used to perform an initial feasibility study to assess the likelihood that a P-DHI will succeed for a specific target group. Using the constructs and factors, it is possible to identify situations in which a P-DHI would most likely succeed or fail and what it would require from the P-DHI in terms of IT and non-IT investments to succeed. For example, success would be difficult in a situation in which the target group has a low socioeconomic status, has a personal life situation characterized by high levels of stress, lacks readiness for health-related behavior change, and inhabits communities that provide little support for healthy behaviors and behavior change, and in which health care organizations lack capabilities in providing services based on the use of P-DHIs and the technological infrastructures in society are unreliable. Textbox 8 describes key questions related to the constructs and factors in the P-DHI investment model that health care organizations should address when considering, designing, and implementing P-DHIs.

**Using the model in practice.**

**Outcome**

- What kind of outcome do we want to achieve for the targeted persons (eg, reducing the risk of cardiovascular disease for a specific target group characterized by a high risk of developing cardiovascular disease)?
- What kind of outcome do we want to achieve for the involved health care organizations (eg, lowering costs and making prevention initiatives easier to access and more attractive for the target group)?

**Context**

- What are the major characteristics of the context?
- In what ways does the context support or hinder the target group’s behavior change and process changes in health care organizations?
- Individual users’ characteristics: the degree to which the preventive digital health intervention (P-DHI) matches individuals’ current stage in the behavior change process and their characteristics and influences these characteristics positively
  Do we attempt to support potential users who have little or no intention of changing their behavior?
  Do we attempt to support potential users who have the intention but need support to successfully change their behavior?
  Do we attempt to support potential users who have succeeded in changing their behavior but need support to maintain the new behaviors?
  What characterizes the potential users, and how should we design the P-DHI to increase the likelihood of adoption and use? How might we support the development of personal characteristics (eg, their awareness about health) in ways that increase the possibilities for success?
- Community-level characteristics: the degree to which the P-DHI supports behavior change in the user’s specific community
  What characterizes the potential users’ communities in terms of typical barriers and resources, and how might we support the users in overcoming barriers and exploiting resources?
  How difficult is it going to be to achieve the behavior change–related outcome for the individual P-DHI users in this community?
- Health care organization readiness: the degree to which the necessary resources are in place to support the implementation of P-DHIs in health care organizations
  Do we have the needed resources for implementing a P-DHI, or do we need to prepare and invest in specific resources before we invest in a specific P-DHI?
  How difficult is it going to be to achieve the outcome for the health care organization?
- Health care sector requirements: the degree to which P-DHIs comply with core health care sector requirements
  Which health care sector requirements do we need to comply with regarding regulation, medical practice, and being evidence-based?
  How are we going to achieve compliance?
- National-level support for the use of P-DHIs: the degree to which national-level funding, policies and regulations, and technological infrastructures support the use of P-DHIs
  What are the possibilities for funding?
  Which policies and regulations (eg, the General Data Protection Regulation) do we need to comply with?
  What characterizes the technological infrastructures that we rely on for the P-DHI, and which limitations and possibilities do they represent?

**Changes**

- What specific changes to the target group’s behaviors represent the easiest way to successfully achieve individual P-DHI users’ outcomes given the context characteristics?
- What specific changes to health care organization processes represent the easiest way to successfully achieve health care organizations’ outcomes given the context characteristics?

**Lag effects**

- When can we realistically expect to experience outcomes from individual-level behavior changes, and which factors drive the lag effects?
- When can we realistically expect to experience outcomes from changes to health care organizations, and which factors drive the lag effects?

**P-DHI IT investment**

- Given the outcomes and changes that we attempt to achieve in these specific contexts, what are the key requirements for the P-DHI apps and how might we address these requirements?
- Complying with software quality requirements: the degree to which the P-DHI app complies with basic software quality requirements and is integrated with other relevant IT systems
  Given the expected outcomes, the behavior changes that we are aiming for, and the requirements we can derive from the context characteristics, how should we define and fulfill the software quality requirements for this specific P-DHI app? For example, what does user friendly mean for this specific app when it is used by these specific users in this specific context?
- Tailored to the context: the degree to which the P-DHI app is tailored to individuals’ context and personal needs
  Given the expected outcomes, the behavior changes that we are aiming for, and the requirements we can derive from the context characteristics, how should we tailor this specific app to make it fit the individual users and their context? What can be achieved through static and dynamic tailoring?
- Developing personal capabilities: the degree to which the P-DHI app supports the development of the personal capabilities needed for behavior change using a P-DHI app
  Given the expected outcomes, the behavior changes that we are aiming for, and the requirements we can derive from the context characteristics, how should we support the P-DHI users in improving relevant capabilities?
- Behavior change support: the degree to which the P-DHI app provides theory-based behavior change support
  Given the expected outcomes, the behavior changes that we are aiming for, and the requirements we can derive from the context characteristics, which model (eg, the transtheoretical model), behavior change techniques, and other theories should we use as the foundation for the design of the P-DHI app? How could we use the theory in the best way?
- Provides additional personal help: the degree to which the P-DHI app provides additional help through access to web-based social networks and health care professionals during the behavior change process
  Given the expected outcomes, the behavior changes that we are aiming for, and the requirements we can derive from the context characteristics, to what extent is personal help from health care professionals needed? How might we use web-based social networks to support the behavior change process? How might we minimize the costs?

**P-DHI non-IT investment**

- Given the outcomes and changes that we attempt to achieve in these specific contexts, what are the key requirements for the P-DHI non-IT investments and how might we address these requirements?
- Integrating the use of P-DHIs into health care organizations: the degree to which the use of P-DHIs is properly integrated into health care organizations’ preventive processes
  Given the outcomes and changes that we attempt to achieve in these specific contexts, what kind of non-IT investments do we need to integrate the P-DHI into the health care organizations’ processes?
- Recruiting and engaging P-DHI users: the degree to which health care organizations succeed in recruiting and engaging users
  Given the outcomes and changes that we attempt to achieve in these specific contexts, how might we recruit and engage potential P-DHI users?
- Providing additional services for P-DHI users: the degree to which health care organizations provide additional services, such as workshops that support behavior change
  Given the outcomes and changes that we attempt to achieve in these specific contexts, what kind of additional services do we need to realize the outcomes?
- P-DHI users’ investments: the degree to which users invest the needed resources in terms of engagement and motivation, time, and money, among others, for changing behaviors
  Given the outcomes and changes that we attempt to achieve in these specific contexts, what and how much do we expect that the P-DHI users need to invest in terms of money, time, engagement, equipment, and other resources to succeed? Can we reduce these investments to make it easier for the P-DHI users?

Using the model, the actual design process starts with deciding which types of tangible and intangible outcomes for the target group and the health care organizations should be offered by this P-DHI (Textbox 8). For example, types of outcomes might be the improvement of individuals’ long-term capabilities for managing their own health, helping individuals achieve short-term outcomes (eg, in terms of reduced body weight within a few weeks), or improving the cost-effectiveness of health care organizations. Deciding on the types of outcomes offered by the P-DHI and understanding how difficult they are to achieve requires insight into the context. Textbox 8 describes key questions that health care organizations need to consider regarding the different parts of the context, for example, the kind of barriers that P-DHI users might experience.

The next step is to identify the easiest means of achieving these outcomes by selecting which individual behaviors and internal processes in health care organizations to change and in which way. Some behaviors may be easier to change than others, and the same applies to organizational processes in health care organizations. The goal is to identify the path of least resistance, that is, identify the set of changes that might achieve these types of outcomes in the easiest way given the insights into individuals in the target group and the health care organizations. The more these behaviors and preventive processes vary across individuals and health care organizations, the more they will require in terms of tailoring possibilities.

The last step is designing the simplest P-DHI—consisting of both IT and non-IT investments—that might achieve these changes. When doing so, it is important to strike the most efficient and effective balance between IT and non-IT investments and consider the lag effects to develop a realistic expectation of when these changes might be accomplished.

The design of a P-DHI using the P-DHI investment model is more comprehensive than simply designing an app as the P-DHI contains both IT and non-IT investments. During the design process, the 5 factors related to the P-DHI app and the 4 factors related to the non-IT investments should be considered, and the design should comply with the requirements that can be deduced based on insights into the context in which it is to be used (Textbox 8).

The design of the non-IT investment includes designing the organizational changes in health care organizations to offer P-DHIs (eg, new policies and ways of working), the implementation process (eg, how to facilitate user acceptance within the organization), the recruitment strategies, and the design of additional services (eg, how to provide training and support). Furthermore, design also includes considerations regarding the personal non-IT investments needed from users for implementing, using, and benefitting from the P-DHI to change their behaviors, for example, when and how they will use the app (eg, how much and how they will use the resources provided by the app), how they will allocate the necessary time and resources and rearrange the immediate context to better support healthy behaviors, and how these investments might be reduced to make it easier to change behaviors.

The model can also be used to evaluate the difficulties and possibilities of implementing P-DHIs in relation to the various aspects of the context and create environments conducive to the use of P-DHIs before investing in a P-DHI as part of a public health initiative. This can be accomplished by reducing barriers and improving supportive resources in the context before the intervention, if possible, or by tailoring the P-DHI to help individuals overcome barriers and exploit resources throughout the intervention.

The model illustrates the complexity and uncertainty related to the use of P-DHIs as a major part of public health initiatives. Developing apps and making them accessible on mobile devices sounds easy; however, developing apps in compliance with the requirements stated by the P-DHI model is quite complicated because of the variety of personal, technical, organizational, and social requirements. Furthermore, implementation is difficult, non-IT investments are considerable, and positive outcomes found in RCT research cannot be assumed to easily translate to large-scale implementations as there is little coordinated control over the factors influencing the outcome. Although some factors may be controlled to an extent by individuals, other factors are under the control of local communities and social networks, health care organizations, government agencies, and private sector companies.

## Discussion

### Principal Findings

The research in this paper set out to amplify our understanding of how to increase the predictability of outcomes from P-DHIs focusing on PA and diet behaviors as well as expand our understanding of how to improve the effectiveness of large-scale implementations. The P-DHI investment model presented in Figure 2 addresses both concerns and helps us understand “under what conditions P-DHIs pay off.”

#### Predictability

The P-DHI investment model can be used to increase predictability in P-DHI research and practice as it includes the major constructs and factors that influence outcomes. The RCT reviews examined in this paper did not include descriptions of the many factors influencing outcomes, which is likely because these descriptions are missing from the individual studies. The reviews typically provide information about the use of BCTs but do not disclose how well the apps support individuals’ capability development or the use of social networks, tailoring, software quality, or the use of general mHealth app quality rating scales [34] and app quality rating scales specifically for health behavior change [81,149]. They do not include information about non-IT investments made by individuals to change behaviors. The RCT research reviewed in this paper provides information about changes in people’s health-related process performance (eg, increased PA) and tangible outcomes for people, such as weight loss. Lag effects were not reported, and the same applies to most factors related to the personal context, with the exception of demographic factors such as age, sex, profession, and health. Community-level contextual factors that influence behavior change (such as social support) were not reported. In addition, factors related to other parts of the context were not reported, but these factors likely do not influence the outcomes of RCT studies. Furthermore, it was not reported how well the constructs were aligned (eg, how well P-DHI apps match individuals and the context in which they live). Similar concerns were raised by RCT reviews emphasizing a need for improved intervention reporting in RCT studies [6,11,14,16,20,150] and for more studies that advance our knowledge on the contribution of the different parts of P-DHIs (eg, BCTs and personal contact with health care professionals) [8,10,11,15,18].

Reporting information about the constructs, the relationships between them, and the categories of factors in the P-DHI investment model when publishing empirical studies would help explain why some P-DHIs fail or succeed for some persons and, thereby, increase outcome predictability and create opportunities for improvement.

#### Large-Scale Effectiveness

The cost-effectiveness of large-scale implementations is reportedly unknown [6], and the literature review conducted in this paper found no information regarding the cost-effectiveness of large-scale implementations. However, the previous section described how the P-DHI investment model can be used during design and implementation to increase large-scale effectiveness.

### Future Research

This research also points toward areas that need further study. There is a need for more empirical research on the contribution of the different parts of P-DHI apps; individuals’ non-IT investments; lag effects; and the many different types of potential outcomes of P-DHI use that extend past the tangible health outcomes, for example, how P-DHIs can be used to increase individual capabilities necessary to experience long-term health benefits. Furthermore, this review identified a need for research that can clarify some of the uncertainties regarding how to best use BCTs and web-based social networks in P-DHIs. Future research could also benefit from including theories from the socioecological tradition to investigate how P-DHIs can not only support individual behavior change but also improve the context in which the behavior takes place. Regarding future literature reviews, the literature review presented in this paper could inspire other researchers to conduct multidisciplinary reviews combining knowledge from different fields. The use of P-DHIs is a multidisciplinary approach, but this does not seem to be reflected in the current research on P-DHIs. The P-DHI model may inspire researchers to address some of the uncertainties raised in this study by exploiting other streams of literature.

### Limitations

This research has limitations related to the way in which the literature review was conducted. The scope of the mHealth implementation literature is broader than that of PA and diet P-DHIs, which introduces the risk that some of the identified factors are less relevant for the PA and diet P-DHIs studied in this review. The argument for using this broader scope is that the major difficulties in implementing these solutions (eg, the existence of supportive policies and infrastructures) are likely independent of the specific types of apps. Another limitation is the breadth of the literature review, which does not cover all the factors in detail. However, the goal was to establish a broad understanding of the constructs and factors influencing outcomes rather than exploring the individual factors in detail. The restricted use of the public health socioecological perspective in the model is another limitation. The reviewed mHealth literature almost exclusively addressed how to support individuals in changing health-related behaviors, but other kinds of mHealth apps with greater focus on changing the context to support healthy behaviors would also add value. Furthermore, there are other literature streams that would be valuable to study to address the research objective, for example, literature on nudging. Finally, the reviewed mHealth RCT research was predominantly based on empirical studies from high-income countries, whereas the mHealth implementation research reviewed was predominantly based on empirical studies from lower-income countries.

### Conclusions

This research suggests that outcome predictability could be improved by including descriptions of the constructs and factors in the P-DHI investment model when reporting empirical studies. Doing so would increase our understanding of when and why P-DHIs succeed or fail. The effectiveness of large-scale implementations may be improved by using the P-DHI investment model to evaluate potential difficulties and possibilities in implementing P-DHIs to create better environments for the use of P-DHIs before investing in them and when designing and implementing them. The cost-effectiveness of large-scale implementations is unknown; implementations are far more complicated than just downloading and using apps, and there is uncertainty accompanying implementations given the lack of coordinated control over the constructs and factors that influence the outcome.

## Appendix

Multimedia Appendix 1 PRISMA-ScR (Preferred Reporting Items for Systematic Reviews and Meta-Analyses extension for Scoping Reviews) checklist.

## Abbreviations

BCT: behavior change technique
mHealth: mobile health
PA: physical activity
P-DHI: preventive digital health intervention
PRISMA-ScR: Preferred Reporting Items for Systematic Reviews and Meta-Analyses extension for Scoping Reviews
RCT: randomized controlled trial
